# Effects of Drying Methods and Temperatures on the Quality of Chestnut Flours

**DOI:** 10.3390/foods11091364

**Published:** 2022-05-08

**Authors:** Veronica Conti, Patrizia Salusti, Marco Romi, Claudio Cantini

**Affiliations:** 1 Department of Life Sciences, University of Siena, 53100 Siena, Italy; marco.romi@unisi.it; 2 National Research Council of Italy, Institute for Bioeconomy (CNR-IBE), 58022 Follonica, Italy; patriziasalusti@libero.it (P.S.); claudio.cantini@ibe.cnr.it (C.C.)

**Keywords:** nutraceutical, antioxidants, polyphenols, volatile compounds, aflatoxins

## Abstract

The demand for chestnut flour is growing because of its use in gluten-free products. Previous studies have correlated the quality of chestnut flours to the drying temperature and technology applied. This work is a novel study on the role of the traditional drying method with a wood fire in a “metato” building for flour compared with a food dryer at 40 °C or 70 °C. The contents of antioxidants, total polyphenols and sugars were determined as well as the presence of toxic volatiles or aflatoxins. The flour, resulting from the traditional method, presented lower polyphenol content and antioxidant power compared to the others. The content of the sugars was similar to the flours obtained after drying with hot air, both at 40 °C and 70 °C. The toxic volatile molecules, furfural, guaiacol, and o-cresol, were found. There was no correlation between the aflatoxin content and the presence of damage in chestnut fruits. The traditional method should not be abandoned since it confers a pleasant smoky taste to the product, but it is necessary to regulate the level and steadiness of temperature. Future research needs to be directed to the quantification of harmful volatile compounds and their correlation with the quantity of smoke emitted by the wood fire.

## 1. Introduction

The cultivation of chestnuts (*Castanea sativa* Mill.) is spread worldwide, especially in Asia and Europe. The European yield of chestnut fruits represents about 12% of the global crop [1]. Italy is the most productive country in Europe (fifth in the world), with nearly 56,000 tons of chestnuts (40% of the European production) harvested on average per year over the period between 1994–2020 [2]. A consistent fraction of Italian chestnuts, approximately 15–20%, are industrially dried and ground into flour [3].

Nowadays, the demand for chestnut and its derivatives is rapidly growing, especially because chestnut flour can be a great substitute for cereal flour and thus a valuable gluten-free alternative [4,5,6,7]. It is, therefore, more and more essential to protect and enhance the quality of chestnut fruits and their transformed sub-products.

Chestnuts are seasonal and present a 50% on a wet basis of water content [8]. One of the best methods of chestnut preservation is drying [9]. Indeed, the drying of agricultural products is a technique that is commonly used to prevent fungal growth [10]. The organoleptic, nutritional, and nutraceutical qualities of chestnuts often depend on the drying temperature, as well as different types of cooking. Roasting, for example, can increase the content of sugars by 25% [11].

Interest has also moved on restoring a traditional drying method called “metato”, a two-level structure, either in stone or brick, where a fire in the ground floor heat chestnuts positioned on the upper floor. Although it is captivating to bring the tradition back into fashion, it is crucial to understand how well this ancient classical method intersects the modern need for product quality and healthiness. Various drying methods can indeed change the product’s characteristics [12]. The quality of the chestnut dried into the “metato” might be harmed by the continuous change in temperature due to the manual managing of the wood fire. Traditional drying could even lead to a high formation of toxic volatile compounds, such as furfural, guaiacol and o-cresol [11,13,14]. The latter two make the product taste smokey, or even burned if present in large amounts, and are formed precisely during the thermal degradation of lignin [9].

Nutraceutical compounds are basic and primary indicators of quality. Chestnuts are good resources of antioxidants, calcium, and polyphenols [15]. Such compounds are now at the center of biomedical studies, apt to define their mechanisms of action at the molecular and cellular levels. Moreover, they should play an important role in the prevention of important chronic diseases [16].

Drying at a low constant temperature is considered the best method to maintain the nutraceutical compounds of foods and this method could be applied also to the chestnut production chain, although most of the producers think that this will completely change the quality of the traditional chestnut flour. Another problem that the traditional method must face, however, is the post-drying selection of the fruits sent to the milling. Once the fruits are dried, they need to be peeled, and only at that moment is it possible to check for the presence of damages, represented by a brown or chalky color of the flesh. These damages could be the result of the activities of insect larvae [17] or fungi, such as *Gnomoniopsis castaneae*, *Sclerotina pseudotuberosa* [18,19], and *Aspergillus* that might produce toxins [20]. When cut open, rotten chestnuts show a tan-brown discoloration of part or the entire nut kernel. Rots can range in color, from chalky-white to dark brown. Likewise, Aspergillus mycotoxigenic fungus may contaminate chestnuts and derived products [21,22]. Aflatoxins are the most toxic products synthesized by *Aspergillus flavus* and *Aspergillus parasiticus*. The main known aflatoxins are named B1, B2, G1, and G2, based on their fluorescence under ultraviolet light (B = Blue, G = Green) [23]. The B1 is the most toxic aflatoxin, classified as a class 1 carcinogen by the International Agency for Research on Cancer (IARC): it is a potent genotoxic carcinogen in laboratory animals and a likely tumorous agent for the human liver [24]. The European Commission (EC) has set the maximum limits for aflatoxins in different foodstuffs. For chestnuts, the maximum levels of B1 and total aflatoxins are 2.0 µg/kg and 4.0 µg/kg, respectively.

The present study reports the results of the comparison between traditional drying using the “metato” comparison to a more efficient and modern method of drying based on steady hot air. For the first time, the effects of the two different drying methods as well as the role of the temperatures on the quality of chestnut flour are evaluated. The antioxidant power, polyphenols, and sugar contents were investigated. The research clarifies whether the aflatoxins content of the chestnut flour can be due only to a scarce selection of the fruits and the presence of rotted chestnuts before the milling. Finally, the study reports the first finding of the presence of o-cresol and guaiacol, potentially harmful compounds in traditional flours.

## 2. Materials and Methods

### 2.1. Collection of Chestnut Flour

The chestnut samples were collected from chestnut plants of the two most widespread cultivars ‘Rossolina’ and ‘Carpinese’ within the Metalliferous hills in Tuscany. Fruits were harvested for two consecutive years in October, the optimal period for fruit ripening. In both years, a homogeneous 100 kg lot of fruits provided by “Associazione Valorizzazione Castagne Alta Maremma” composed of a 50% weight of “Rossolina” and 50% of “Carpinese” was selected to represent the mean composition of the crop in the area. The lot was then divided into three sub-samples of 5 kg each. The sub-samples were then dried with three different methods:

- By the traditional “metato” drier, working with a wood fire, covered by ashes, where the fluctuating temperature recorded by a data logger localized into the fruit container ranged from a minimum of 30 °C to a maximum of 70 °C, with a mean value of 37 °C;
- By a laboratory oven (Biosec, Tauro, Camisano Vicentino, Italy) at a constant temperature of 70 °C;
- By a commercial air dessicator (Biosec, Tauro, Camisano Vicentino, Italy) at a steady temperature of 40 °C.

The “metato” is usually started when it is completely filled with chestnuts, forming a layer of about 50 cm in height which is daily moved by hands. To separate each sample from the mass, the chestnuts were inserted into a large bag made of natural fibers which do not modify the air fluxes through the fruits. This method is also used when a small producer needs to have only its part of chestnuts back. The drying in the “metato” took 40 days in year 1 and 35 days in year 2. This was the period the producer’s association used for the production of the mass flour to be commercialized.

The dried chestnuts, resulting from each method, were then manually peeled and sorted to be classified as follows:

- Non-defective: any color alteration was present neither outside nor inside, with the chestnut appearing totally white;
- Defective: alterations present in 25 ± 2% of the volume of the nuts with the flesh turned brown/chalky white.

The percentage of the alteration was determined by taking a picture of the fruits and measuring the different areas (good and rotted) using the ImageJ program, and then selecting only the chestnuts with about 25% of the external surface interested by a change in color (percentage chosen on the basis of Morales-Rodriguez et al. (2022) [25]. This level was chosen because it is easily scorable by manual sorting and it could correspond to the maximum level of defect that a producer could leave in the worst case. It was not possible to use the weight of the rotted and healthy portions because they presented different specific weights. Before the milling, the nuts were manually cut, and we confirmed that a superficial alteration showed the presence also of internal rotting.

Finally, the dried chestnuts were ground to flour using an electric stone mill (Jumbo model, KoMo Gmbh & Co., Ltd., Hopfgarten, Austria), positioning the selector for the maximum refinement of flour. Summing up, 12 flour samples were obtained according to the different drying methods (3), chestnut state (2), and year of harvest (2).

### 2.2. Moisture Determination

The moisture content was measured using the official Italian method for food moisture determination described by the Istituto Superiore di Sanità (G. U. n. 145 21/6/1985) [26]: 10 g of each sample was put on a weighing filter and introduced into an oven set at 103 °C for 12 h. The sample was then left to cool down, and the weight was checked until it reached steadiness. The moisture of the sample was calculated on the base of the final and initial dry mass and expressed as a percentage.

### 2.3. Sample Extraction for Colorimetric Assays

For each triplicate sample, 9 mL of 70% acetone was added immediately to 0.5 g of flour [27]. The tube was then rapidly homogenized by an Ultra-Turrax^®^ T-25 basic (IKA^®^-Werke GmbH & Co. KG, Staufen im Breisgau, Germany) and sonicated for 20 min with an Elma Transsonic T 460/H. Finally, the sample was centrifuged for 5 min at 1500 RCF (Eppendorf^®^ Microcentrifuge 5415D, Hamburg, Germany).

### 2.4. Determination of Antioxidant Power

The ferric ion reducing antioxidant power (FRAP) method was used to determine antioxidant power [28]. For each reaction, 2040 µL of 300 mM acetate buffer pH 3.6 (consisting of acetic acid and sodium acetate), 200 µL of 10 mM TPTZ (2,4,6-tripyridy-s-triazine), 200 µL of 20 mM ferric chloride (FeCl_3_) and 20 µL of extract (distilled water for white) were mixed. After, the mix was incubated at 37 °C for 1 h and the absorbance was read at 593 nm using a Double beam UV/Visible spectrophotometer (Perkin Elmer, Waltham, MA, USA). The absorbance value was interpolated with the standard curve related to ferrous sulfate. Values were expressed in µmol of ferrous (Fe^2+^) equivalent per g of flour and in the form of mean ± standard deviation.

### 2.5. Determination Phenolic Content

The Folin–Ciocâlteu method measures the content of total polyphenols [29]. For each reaction, 500 µL of extract (distilled water for white), 3950 µL of distilled water, 250 µL of F-C reagent (Sigma Chemical, St. Louis, MO, USA) and 750 µL of a sodium carbonate saturated solution (Na_2_CO_3_) were mixed. This mix was incubated at 37 °C for 30 min. The absorbance values were read at 795 nm by a Double beam UV/Visible spectrophotometer (Perkin Elmer, Waltham, MA, USA). The data were then processed through a calibration curve of gallic acid (Sigma Chemical, St. Louis, MO, USA) previously constructed. The total phenolic content was expressed in mg of gallic acid equivalents (GAE) per g of flour and in the form of mean ± standard deviation.

### 2.6. Analysis of Sugars

High-pressure liquid chromatography (HPLC Waters system MOD 2410 refractive index detector, equipped with a 600E pump and thermostating oven at 90 °C, Marshall Scientific, Hampton, NH, USA) was used for the analysis of the sugar components sucrose, fructose, glucose, galactose, and mannitol. Briefly, 100 mg of flour was added to 1 mL of dH_2_O. Samples were homogenized by Ultra-Turrax^®^ T-25 basic (IKA^®^-Werke GmbH & Co. KG, Staufen im Breisgau, Germany), centrifuged at 3000 RCF for 5 min, the supernatants transferred to 2 mL Eppendorf^®^ tubes and then centrifuged again at 12,000 RCF for 5 min (Eppendorf^®^ Microcentrifuge 5415D, Hamburg, Germany). Samples were filtered (0.45 µm) and 20 µL of each extract was injected and examined using a Waters Sugar-Pak I ion exchange column (6.5 mm × 300 mm) at a temperature of 90 °C. The mobile phase consisted of MilliQ H_2_O (pH 7) with a flow of 0.3 mL min^−1^. The overall duration of the separation was 30 min. Identification of the components was obtained using a Waters 2410 refractive index detector, by comparing the retention times with those of the reference standards.

### 2.7. Aflatoxin Content Determination

The aflatoxin content analysis, regulated by international methods, was conducted by a certificated private laboratory (Ecogam, Grosseto, Italy). For each flour, aflatoxins B1, B2, G1, and G2 were quantified using high-performance liquid chromatography (HPLC).

### 2.8. Toxic Volatile Compounds by SPME-GC-MS

For each sample of chestnut flour, 1 g was homogenized with 2 mL of dH_2_O and 1 g of NaCl by Ultra-Turrax^®^ T-25 basic (IKA^®^-Werke GmbH & Co. KG, Staufen im Breisgau, Germany) for 3 min. The entire procedure was carried out under ice at 4 °C. The volatile compound profile was obtained with the SPME-GC-MS technique, following Pugliese et al. [30]. The analysis was performed using an Agilent 7890 GC chromatograph equipped with a 5975A MSD with EI ionization. In the headspace of the vials, a three-phase DVB/Carboxen/PDMS 75-µm SPME fiber was exposed, (Supelco, Bellafonte, PA, USA) at 60 °C for 30 min for volatile compound sampling after 5 min equilibration time. To ensure consistent SPME extraction conditions, a Gerstel MPS2 XL autosampler, equipped with a magnetic transportation adapter and a temperature-controlled agitator (250 rpm with on/cycles of 10 s), was used. The chromatographic conditions were: a column J&W Innovax 30 m, 0.25 mm, ID 0.5 µm DF; injection temperature 250 °C, splitless mode, oven program 40 degrees for 1 min then 2 °C/min to 60 °C, then 3 °C/min to 150 °C, then 10 °C/min to 200 °C, then 25 °C/min to 260 °C for 6.6 min. Mass spectra were acquired within the M/Z interval 29–350 with an Agilent 5975C MSD spectrometer at a scan speed to obtain three scans per second/s. The various volatile compounds were then identified using the MassHunter Qualitative Analysis GC/MS (Agilent) software and were subsequently confirmed by the NIST MS search 2.2 database.

### 2.9. Statistics

The experiments were conducted in three technical replicates for each sample. Finally, the mean and standard deviation were calculated. Statistical *t*-Student tests were performed to highlight the significant differences among treatments (*p*-value ≤ 0.01).

## 3. Results and Discussion

### 3.1. Moisture Determination

**Figure 1:**
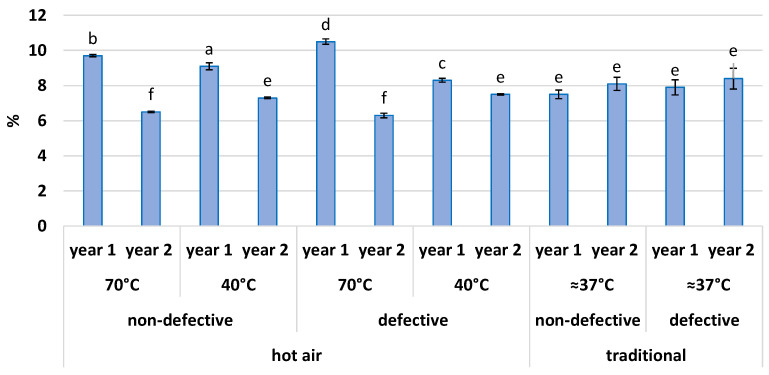
Moisture of chestnut flours samples expressed in percentage. The bars represent mean ± standard deviation. The columns are grouped by drying method, drying temperature (°C), health condition of fruits, and year. Each different letter corresponds to a significant difference with a *p*-value ≤ 0.01.

Even though the conservation and stability of flour depend also on moisture content, there is currently no specific law regulating the maximum allowed for chestnut flour. The production regulations of protected designation of origin (PDO) chestnut flours are the only available references. The Garfagnana and Lunigiana PDO flours have approximate moisture contents of 13% [31] and 8% [32], respectively, corresponding to an average value of 10.5%. However, it is also possible to rely on the existing limits for cereal flours. For the latter, the Italian law nr. 580 of 1967 art. 7 sets an upper limit for the moisture at 14.5% [33]. In Figure 1, the percentage of moisture measured each year and for each sample of flour is reported. Although with differences among the samples, the level of moisture for each flour was below the Italian legal limitation for cereals and, in general, comparable to or lower than the typical values of PDO chestnut flours. Thus, all the flours started from an excellent drying state.

### 3.2. Determination of Antioxidant Power

**Figure 2:**
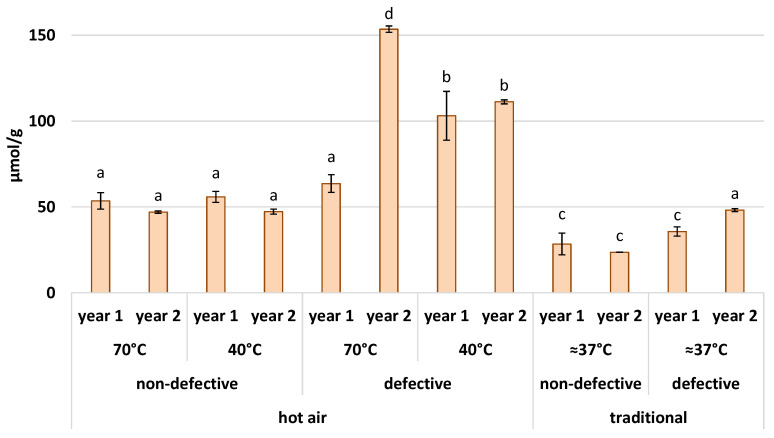
Antioxidant power of chestnut flours in µmol (Fe^2+^E)/g ± standard deviation. The columns are grouped by drying method, drying temperature (°C), health condition of fruits, and year. Each different letter corresponds to a significant difference with a *p*-value ≤ 0.01.

The antioxidant power indicates the ability of antioxidants of a fruit or a substance to scavenge ROS and thus is a key index of nutraceutical properties [34]. In this study, the FRAP method was performed to get an analytical and comparable measure of the antioxidant power. In Figure 2, the results for each flour sample are shown. The non-defective flours have an antioxidant content that ranges from 23.671 µmol/g (traditionally dried flour in year 2) to 55.890 µmol/g (dried at 40 °C hot air in year 1), indicating a marked influence of the drying method on this variable. Other previous studies, indeed, demonstrated that the drying temperature affects the morphological and chemical properties of chestnut flours [1], and in rice paddy herbs there are differences in phenolic compounds and antioxidant properties as well [35]. In our study, if only non-defective flours are considered, the chestnut flours dried with hot air at 70 °C and 40 °C, for both years, present higher values at around 50 µmol/g. In the case of flours produced with fruits with the presence of color changes due to damages caused by parasites, the values are significantly higher. These flours have an antioxidant power that ranges from 35.710 µmol/g (traditionally dried flour in year 1) to 153.508 µmol/g (dried with hot air at 70 °C in year 2). This behavior is probably due to the damage induced by biotic or abiotic stress, which often leads to an increase in ROS. A method to scavenge these free radicals is with the production of antioxidant compounds, which compensates for the damage [36]. In fact, biotic stresses lead to the formation of ROS, which also acts as the first signal for gene activation that allows the plant to respond to the pathogen. Among these responses, secondary metabolites useful for scavenging excess ROS are also synthesized [37].

### 3.3. Determination Phenolic Content

Polyphenols are the most representative antioxidant molecules in the vegetable kingdom and their content is one of the main nutraceutical attributes of food [38]. The Folin–Ciocâlteu method allowed us to identify the total polyphenol content with precision and to compare the different flour samples.

**Figure 3:**
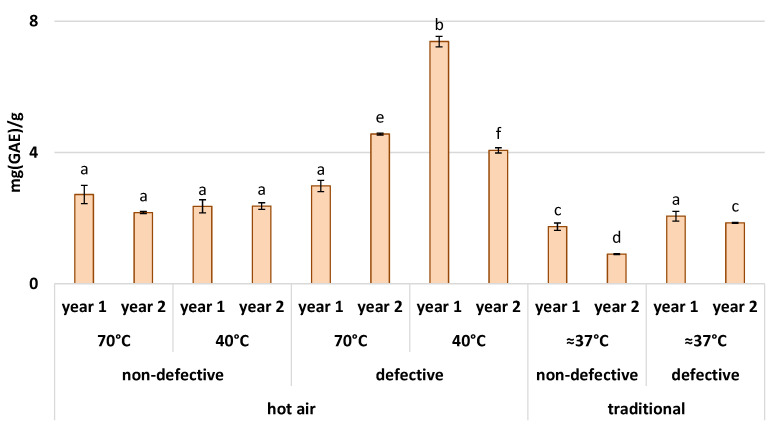
Polyphenol content (mg(GAE)/g ± standard deviation) of chestnut flours. The columns are grouped by drying method, drying temperature (°C), health condition of fruits, and year. Each different letter corresponds to a significant difference with a *p*-value ≤ 0.01.

In Figure 3, the polyphenol content measured for each flour is shown. Considering only the non-defective flours, the values vary from a minimum of 0.940 mg/g for traditionally dried flour in year 2, to a maximum of 2.720 mg/g for hot air-dried flour at 70 °C in year 2. As for the antioxidant power, the values of polyphenol content change according to the type of drying and are higher for the flours derived from air-dried chestnuts. In general, according to a similar study by Piga et al. (2003) [39] on plums with constant drying temperatures, the polyphenol content depends on the drying temperature, although each type of polyphenol can be affected differently. It is difficult to relate the decrease in polyphenols as the one reported in Figure 3 with a different activity of enzymes that promote the oxidation of polyphenols, such as polyphenol oxidase (PPO). For example, PPO’s action can differ with the type of polyphenol, while in this study, only the total polyphenol content was examined. This also depends on the drying temperature [39], which, in the traditional method, could oscillate rapidly from a minimum of 30 °C to peaks of 71 °C. Therefore, the results in this study can only point out that the oscillatory behavior of the temperature in the traditional method leads to a decrease in the polyphenolic content more prominently than in the case of hot-air drying, where, instead, the temperature is kept constant. Regarding the flours derived from defective chestnuts, the content varies between 1.857 mg/g (traditionally dried flour in year 2) and 7.380 mg/g (dried with hot air at 40 °C in year 1). As for the antioxidant power, the polyphenol content is again higher for defective flours. In fact, polyphenols, and, in general, antioxidants, are part of the plant’s secondary metabolism, which is most useful for defense against various stresses, such as parasites and insects [40]. In this case, the chestnuts were probably attacked by larvae of *Cydia Splendana* and *Circulio elephas* or by mold and fungi. These attacks are seen as biotic stress and stimulate the production of secondary defense metabolites. The latter, in fact, promotes the scavenging of ROS produced by the attack of the pathogen [37].

### 3.4. Analyses of Sugars

**Figure 4:**
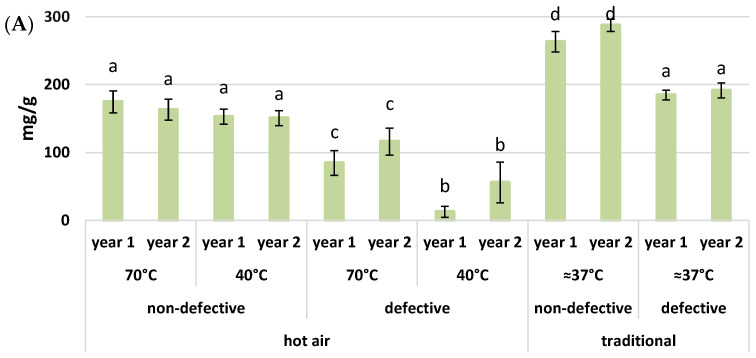

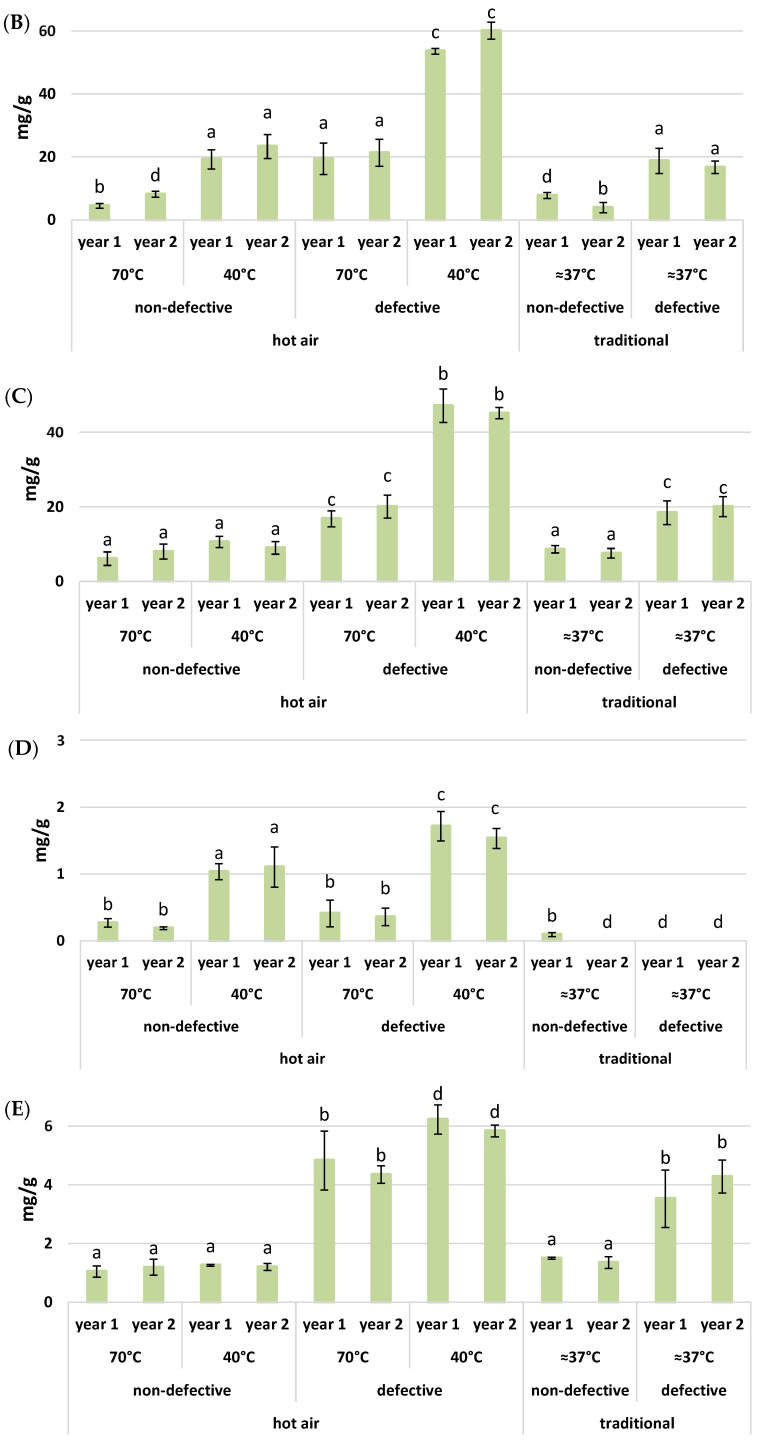
Sugar content (mg/g) in chestnut flours is grouped on the basis of drying method, chestnut condition (non-defective and defective), temperature (°C), and year. (**A**) sucrose; (**B**) glucose; (**C**) fructose; (**D**) galactose; (**E**) mannitol. Each different letter corresponds to a significant difference with a *p*-value ≤ 0.01.

The drying temperature affects the quantity of sugars [13]. The variation of sugars (sucrose, glucose, fructose, galactose, and mannitol) is due to the hydrolysis of starch, the decomposition of sucrose into glucose and fructose, and the caramelization and degradation of sugars [41]. The results obtained and depicted in Figure 4 reveal a slight increase in sucrose (Figure 4A) for the flours dried at 70 °C compared to those at 40 °C, and it is higher in flours dried with the traditional method, where the temperature was unstable, in agreement with Piccolo et al. [42]. This behavior is also in line with the results by Carreia et al. [1], who concluded that the higher the drying temperature is, the higher the sugar content, and in particular, sucrose. The latter indeed increases with temperature because of the thermal and enzymatic degradation. The reason for glucose and fructose rising is yet unknown. In any case, the behavior of glucose, fructose, and galactose is different, as can be seen in Figure 4B–D. The highest content appears for the dried flour at 40 °C. For traditionally dried flours, there is just no trace of galactose.

What is also noted, however, is an increase in sugars (glucose, fructose, galactose, and mannitol) in the defective flours. In fact, sugar content typically increases when the plant or fruit is under stress [43]. The stressful conditions seem to favor the accumulation of fructose and glucose that results from the degradation of sucrose as a response to stress [44]. Mannitol contributes to the osmotic potential and plays a major role in the abiotic stress response mechanism [45,46]. Therefore, the increase resulting in the present work (Figure 4E) is likely due to a response to oxidative stress and an accumulation of ROS. In fact, sugars are, in general, involved in the defense response, as they serve as precursors of various secondary metabolites [47,48,49].

### 3.5. Aflatoxin Determination

The aflatoxin content might help to obtain a general picture of the quality of a product. Several studies reported the presence of *Aspergiullus flavus* and the contamination from aflatoxin in chestnuts and derivates [3,10,21]. Drying temperature and storage can affect aflatoxin production by mycotoxic fungi, as demonstrated by the study on hazelnuts where aflatoxins were not recorded at higher drying temperatures (above 40 °C), even after storage at 25 °C [50]. The total aflatoxins are largely within the legal content in each of the analyzed flour. For B2, G1, and G2 aflatoxins, the values are below the limit of detection (LOD), namely, they are less than 0.5 µg/kg.

Concerning B1 aflatoxin, all the flours are within the allowed limits. Hence, there is no correlation between the defective chestnuts and toxicity in terms of aflatoxins content. In fact, in the case of hot air drying at 40 °C in the first year, the content of B1 is higher in non-defective flours with respect to defective ones.

### 3.6. Toxic Volatile Compounds by SPME-GC-MS

**Figure 5:**
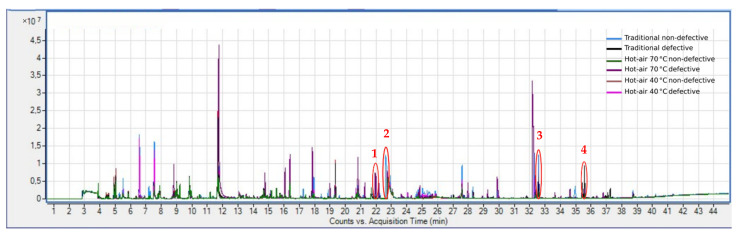
Chromatograms of 8 flours (representative of 2 years) distinguishable with different colors: light blue and blue for traditionally dried non-defective, black for traditionally dried defective, green and red for 70 °C hot air-dried non-defective, purple for 70 °C hot air-dried defective, brown for 40 °C hot air-dried non-defective and pink for 40 °C hot air-dried defective. The relevant peaks are circled and numbered from 1 to 4: **1** for 1-octe-3-ol, **2** for furfural, **3** for guaiacol (phenol, 2-methoxy), and **4** for o-cresol (phenol, 2-methyl).

The type of cooking and drying can lead to the formation of toxic volatile compounds [11]. In cooked chestnuts, aromatic components have often been found to derive from the degradation and caramelization of saccharides, degradation of proteins and lipids, and the Maillard reaction [13]. An example of these compounds is furfural, which is usually associated with hints of caramel, roasted coffee, and baked bread [51]. In Figure 5, the furfural peak appears in the profile of traditionally dried non-defective flour (light blue chromatogram, peak number 2). This compound is commonly present in many foods but becomes toxic if it exceeds certain quantities (in mice and rats, for example, it has been found to be toxic with LD50 of 400–500 mg/kg and LD50 of 50–149 mg/kg, respectively) [52]. The present study detects the presence of furfural in all the samples and, although not absolutely quantified, the peak for the traditionally dried non-defective flour presents an area that is more than three times larger than the average and it is significative different (Table 1). 

Other compounds typically found in traditionally dried flours are guaiacol (phenol, 2-methoxy) and o-cresol (phenol, 2-methyl), which are formed during the thermal degradation of lignin [9]. These two compounds can be perceived by consumers as a smoky aroma or, when in high concentrations, associated with the smell of burning [9]. There is evidence that cresols, as well as guaiacol, when reaching high concentrations, can be toxic (for guaiacol there is toxicity in rats with LD50 of 520 mg/kg, while for o-cresol there is acute toxicity in mice and rats with LD50 of 344 mg/kg and 121 mg/kg, respectively) [14,53]. The o-cresol was found in traditionally dried defective flour (black chromatogram), while guaiacol was in traditionally dried non-defective flour (light blue chromatogram). Even in this case, the peak of both o-cresol (peak number 4) and guaiacol (peak number 3) is more than twice larger than the average of all flours and these are significative different.

Another relevant substance is 1-octen-3-ol, which is commonly associated with the fungal aroma [51] and has shown some toxicity in tests on human cells [54]. This compound is found in 70 °C hot air-dried defective flour (purple chromatogram) and therefore derived from visibly defective and moldy chestnuts. The area of the peak (peak number 1) is more than four times larger than the average area of all the flours and it is significative different. As for the flours dried with 40 °C hot air and for 70 °C hot air non-defective, there are no peaks of interest from a toxicological point of view. Ethanol (retention time: 7.5 min) is found only in defective flour, perhaps a sign of alcoholic fermentation due to molds.

## 4. Conclusions

The aim of the present study is to evaluate the effect of drying methods and temperature on the chemical and health quality of chestnut flour. Based on the results obtained, it is possible to conclude that the flour produced with fruits dried with woody fire by the traditional “metato” method has a polyphenol content and an antioxidant power lower than the other flours. This is probably due to the highly-fluctuating temperature behavior of the traditional method. Conversely, in the flours dried with hot air at a constant temperature of 40 °C or 70 °C, the polyphenol content is kept higher. In fact, the greater or lesser activity of enzymes that promote the oxidation of polyphenols may depend on the temperature and the stability to which they are subjected. The content of sugars is similar to the flours obtained by hot air, both at 40 °C and 70 °C. On the contrary, the traditionally dried flour shows a total absence of galactose. Furthermore, traditionally dried flours contain some potentially toxic volatile molecules (furfural, guaiacol, and o-cresol). This rings the first alarm bell, since the presence of such compounds in high concentrations can be toxic to human health. In the future, to establish the toxicity level of chestnut flours, more detailed analyses on the exact content of toxic compounds could be performed. For the parasitized flours, the content of sugars and biomolecules is higher with respect to the healthy ones. Polyphenols and antioxidants in general are secondary metabolites for plant defense and sugars often act as substrates for the latter. Hence, in adverse conditions, the production of these metabolites is enhanced. Despite being beyond the scope of the present study, the aroma of the parasitized flours highlights the presence of a musty character; therefore, the positive higher content in polyphenols does not correspond to a likewise positive organoleptic profile. From the obtained results, however, it emerges that there is no evident correlation between the aflatoxin content in flours and the presence of damages caused by various parasites in chestnut fruits (all flours are within the permitted values). In the end, the most evident result of this work is that a constant high temperature of drying leads to a better-quality chestnut flour. Moreover, the traditional method should not be discarded, although there is a need for a more modern approach oriented to regulating the level and steadiness of temperature during the drying process. Ultimately, volatiles (the presence and type of which depend on the drying method) can confer a pleasant aroma to chestnut flours, this being a positive property if their content is below the toxicity threshold. For instance, 1-octen-3-ol gives a mushroom-like and earthy green aroma, while guaiacol confers a pleasant smoky one. Further research might be directed to the exact quantification of harmful and pleasant volatile compounds and their correlation with the method of drying or the quantity of smoke emitted by the wood fire.

## Figures and Tables

**Table 1:** The retention time (RT) and area of four molecules detected in examined flours in Figure 5. The values in bold correspond to the largest areas of each column. Asterisks indicate a significative difference (*p*-value ≤ 0.01) from the mean of the areas of all flours for each relevant peak.

	1-Octen-3-ol		Furfural		Guaiacol		O-Cresol	
	RT	Area	RT	Area	RT	Area	RT	Area
Traditional non-defective	22.003	1,298,711	22.926	**3,288,748** *	32.584	**8,686,673** *	35.439	836,714
Traditional defective	22.001	640,837	22.922	53,749	32.582	4,408,542	35.435	**2,602,744** *
Hot air 70 °C non-defective	21.999	431,202	22.922	438,989	32.584	1,097,308	35.437	334,842
Hot air 40 °C defective	22.003	**5,320,343** *	22.924	585,345	32.586	678,755	35.435	177,180
Hot air 70 °C non-defective	22.001	259,093	22.926	251,179	32.586	2,334,425	35.435	728,489
Hot air 40 °C defective	22.001	609,126	22.928	1,590,987	32.584	3,752,913	35.435	1,416,660

## Data Availability

No new data were created or analyzed in this study. Data sharing is not applicable to this article.
